# How Disease Is Spread

**Published:** 1895-08-31

**Authors:** 


### The Hospital

AN INSTITUTIONAL JOURNAL OF

The Medical Sciences and Hospital Administration,

WITH

AN EXTRA NURSING SUPPLEMENT.

VOL. XVIII.—No. 466.] [AUG. 31, 1895.

### HOW DISEASE IS SPREAD.

WALTON-ON-THE-NAZE presents to the holiday-making public at the present time an object-lesson which it is to be feared has many parallels. For the reason that there are so many places which do exactly as Walton-on-the Naze is now doing when opportunity offers, it is desirable that the veil should be lifted from the comparative obscurity of Walton, and that that little seaside place should be exposed to the gaze of those who, in seeking change and recreation after many months of toil, so often fall, not "among thieves," but among those who may prove to be very much worse than thieves.

Walton-on-the-Naze, writes a correspondent of the *Times*, is at the present moment passing through an epidemic of diphtheria; and yet nobody in Walton seems to know anything about it. According to a local newspaper, indeed, it would appear that the town is exceptionally healthy; for, as the paper informs its readers, Dr. W. E. Dawson "wishes emphatically to state that he has not had a case of infectious disease of any kind at Walton-on-the-Naze during the last twelve months." All this is, no doubt, quite true so far as Dr. Dawson is personally concerned. He is not, however, the medical officer of health for the district, that post being filled by Mr. A. S. Ivens, whose residence is at Thorpe-le-Soken, a considerable distance away. Mr. Ivens, on his part, has not thought it desirable to publicly deny the existence of a series of cases of diphtheria at Walton-on-the-Naze.

But, in truth, the evidence furnished by the correspondent of the *Times* leaves no manner of doubt as to the facts of the case. The way in which the epidemic was discovered is worthy of note. An active school visitor, issuing summonses for the non-attendance of children at school, finds that two or three have died of diphtheria, and that several others are down with it. In all the officer discovers nine cases, with two or three deaths. Nine cases do not make an epidemic, it may be said. But in a small place like Walton-on-the-Naze, with a population of less than sixteen hundred, nine cases do make a very decided epidemic—an epidemic so alarming that if we had a similar proportion of diphtheria cases to population in London, every morning and evening newspaper would be full of the subject, and every family which could afford to live out of London for a few weeks would promptly take to flight. Nine cases at Walton are equivalent to more than 5 per 1,000. If diphtheria prevailed in London to exactly the same extent, there would now be no fewer than 25,000 cases in the whole metropolis, an epidemic truly appalling.

The shameful feature of the case is that whilst Walton-on-the-Naze is the subject of an epidemic of diphtheria in the midst of the visiting season, there is no attempt made at suppression, nor, so far as we know, even at isolation; but, on the contrary, a very determined effort is made to hush the matter up in order that as many strangers as possible may be lured to the locality of the deadly pestilence. If there are nine known cases there may be more that are unknown. Each case is a focus of radiation for the disease. Walton is a town which is popular among church choirs and Sunday-school children. Steamers from London discharge their cargoes of passengers at Clacton, and many of these go direct to Walton as their ultimate objective. In short, it is easy to imagine that if the epidemic had been one of small-pox or cholera instead of diphtheria it might by this time have been spread over the greater part of England.

The average holiday-maker stands aghast at such revelations as these. Have I no remedy, he asks, if I go to Walton and am prostrated by diphtheria? Are there any other towns which behave in the same wicked and shortsighted fashion? Is it possible to punish such towns for their cold-blooded disregard of everything but their own pecuniary interests? These are the questions which hundreds of persons in this holiday season are now asking. As a matter of fact there is not, so far as we know, any law by which such towns as Walton can be brought to book for any ills which their short-sighted selfishness may bring upon the country at large. But there is a rod of correction for such towns, and it ought to be unsparingly used. They must be boycotted one and all. Nothing brings places of summer resort so rapidly to their senses as a determined application of the boycott. Let them be left to themselves; let their lodgings stand empty, and their shops be left without customers.

One or two seasons of this remedy are generally quite sufficient to bring the meanest ratepayer to a sense of his duty to the public. Health resorts are of the most prime importance to a strenuously toiling generation. To turn such places into death-traps in order that a few pence per head may be saved on the rates is from the point of view of the places themselves nothing short of imbecility, whilst from the standpoint of the holiday-seeking public it is a crime to be punished with the utmost available severity. Whatever happens to the pockets of tradesmen and lodging-house keepers in health resorts the public must protect itself. If the inhabitants of such places desire to be permanently prosperous they must above all things make their places "health resorts" in fact as well as inname.

